# Impaired glucagon suppression and reduced insulin sensitivity in subjects with prediabetes undergoing atorvastatin therapy

**DOI:** 10.1530/EJE-19-0173

**Published:** 2019-09-23

**Authors:** Francesca Urbano, Antonino Di Pino, Roberto Scicali, Agnese Filippello, Stefania Di Mauro, Alessandra Scamporrino, Simona Marchisello, Agata Maria Rabuazzo, Francesco Purrello, Salvatore Piro

**Affiliations:** 1 Department of Clinical and Experimental Medicine, Internal Medicine, Garibaldi-Nesima Hospital, University of Catania, Catania, Italy

## Abstract

**Objective:**

Statin therapy has been linked to an increased risk of type 2 diabetes in high-risk populations; however, the pathophysiology of this association remains to be clarified. We investigated glucagon suppression and its relationship with insulin resistance in prediabetic subjects undergoing atorvastatin therapy; in addition, we studied molecular insulin signaling in pancreatic α-cells exposed to atorvastatin *in vitro*.

**Design and methods:**

Fifty subjects with prediabetes were divided into two groups based on atorvastatin therapy. All subjects underwent an oral glucose tolerance test. Early (0–30 min), late (30–120 min) and overall (0–120 min) glucagon suppression were evaluated. Insulin sensitivity was estimated by the insulin sensitivity index (ISI_0–120_). Insulin signaling pathway and insulin-mediated glucagon suppression were investigated in pancreatic αTC1-6 cells chronically exposed (24 or 48 h) to atorvastatin (100 ng/mL).

**Results:**

Individuals on statin therapy (*n* = 26) showed a significantly reduced early (0–30 min) (*P *= 0.003) and overall (0–120 min) (*P* = 0.01) glucagon suppression compared with controls (*n* = 24). In multivariate regression analysis, early glucagon suppression (0–30 min) exhibited a significant correlation with statin therapy. Regression analysis showed a significant association between ISI _0-120_ and early_0-30_ (*r* = 0.33, *P *< 0.05) and overall_0_-_120_ (*r* = 0.38, *P *< 0.05) glucagon suppression. Moreover, in αTC1-6 cells atorvastatin treatment affected insulin-mediated glucagon suppression, insulin receptor phosphorylation and IRS-1-AKT pathway signaling.

**Conclusions:**

Prediabetic patients undergoing statin therapy exhibit impaired glucagon suppression associated with lower insulin sensitivity. Our data revealed a new molecular aspect behind the deregulation of insulin sensitivity secondary to statin exposure.

## Introduction

Statins, hydroxymethylglutaryl-coenzyme-A (HMG‑CoA) reductase inhibitors, are first-line agents for the management of hyperlipidemia in patients at high risk of cardiovascular (CV) disease and are one of the most commonly prescribed CV drugs worldwide (1, 2).

Although statins have proved to be generally well tolerated with a low prevalence of adverse effects, over the past decade, a growing body of evidence has linked chronic statin therapy to an increased risk of new-onset diabetes mellitus (NODM) (3, 4, 5); these findings were supported by observational studies (6, 7, 8, 9), controlled clinical trials (10, 11), and meta-analyses (12, 13, 14, 15, 16). People with pre-existing risk factors for diabetes (such as prediabetes) seem to more likely to develop statin-mediated type 2 diabetes than those without (17); therefore, baseline prediabetes or metabolic syndrome components could be of predictive importance for NODM in subjects treated with statins. Benefits of statins in reducing cardiovascular events far outweigh the risk of diabetes (10, 18, 19); nevertheless, it might be relevant to elucidate the molecular mechanisms at the basis of the relationship between statin therapy and increased diabetes risk.

It is known that impaired pancreatic β-cell secretory function and insulin resistance are the primary pathologic abnormalities in patients with type 2 diabetes and both of these mechanisms have been proposed to be involved in NODM secondary to statin treatment (20). We previously showed that insulin secretion from β-cells is inhibited by chronic exposure to atorvastatin and that this secretory alteration is associated with mitochondrial dysfunctions induced by conditions of oxidative stress (21). Moreover, other studies have reported that insulin sensitivity and expression of GLUT4 were impaired in 3T3-L1 adipocytes treated with lovastatin (22).

In addition to β-cell secretory failure and insulin resistance, also α-cell function is essential for glucose regulation both in fasting and postprandial state; indeed, patients with type 2 diabetes frequently manifest hyperglucagonemia that contributes to uncontrolled hyperglycemia (23, 24, 25).

The secretion of glucagon is complex and involves a combination of various factors (26); defective suppression of glucagon by glucose or insulin and especially insulin resistance in α-cells have been suggested as potential mechanisms for the hyperglucagonemia in type 2 diabetes (27). In addition, we and others have reported that insulin signaling in α-cells plays a critical role in the regulation of glucagon secretion and that impaired insulin signaling leads α-cells to develop a diabetic phenotype due to enhanced glucagon secretion (28, 29, 30).

Hyperglucagonemia, failure to suppress glucagon and increased insulin resistance could contribute to the impaired glucose regulation in subjects on statin therapy. To date, no studies have investigated the role of α-cells in patients at high risk for type 2 diabetes treated with statins.

In the present study, we examined plasma glucagon concentrations and glucagon suppression after oral glucose intake in a group of individuals at high risk for type 2 diabetes (prediabetes) undergoing atorvastatin therapy. Additionally, to further explore the effect of statins on α-cell function, we investigated insulin-mediated glucagon suppression at the cellular and molecular level using an *in vitro* model of α-cells (αTC1-6) chronically exposed to atorvastatin.

## Subjects and methods

### Participants

This was a cross-sectional study investigating glucagon suppression and its relationship with insulin resistance in prediabetic subjects undergoing atorvastatin therapy.

This study was conducted on fifty subjects attending our University Hospital for metabolic risk evaluation. Subjects were screened at the Day Service of Internal Medicine Division at the Garibaldi Hospital in Catania, Italy from January 2016 to January 2017.

The following inclusion criteria were applied: age range between 35 and 65 years; body mass index (BMI) between 18.5 and 40 kg/m^2^; on statin therapy (atorvastatin 40 mg for at least 5 years); prediabetes identified according to the American Diabetes Association (ADA) criteria (impaired fasting glucose and/or impaired glucose tolerance, and/or glycated hemoglobin (HbA_1c_) between 5.7 and 6.4%) and Caucasian race.

The exclusion criteria were a previous history of diabetes, a previous history of overt cardiovascular events (stroke, ischemic heart disease, chronic obstructive peripheral arteriopathy or heart failure), anemia or hemoglobinopathies, the use of medications known to affect glucose metabolism, clinical evidence of liver or renal disease, chronic diseases, and/or recent history of acute illness, malignant disease, and drug or alcohol abuse.

Each of the prediabetic patients on statin therapy was matched to one or more controls. Controls were identified from subjects with prediabetes without previous statin therapy attending our University Hospital for metabolic risk evaluation and were matched to the patients with statin therapy by age, sex and BMI. The goal of matching was to increase the study’s efficiency by forcing the case and control samples to have similar distributions across confounding variables.

### Biochemical analysis

After overnight fasting venous blood samples were obtained for the measurement of biochemical parameters. Serum total cholesterol, triglycerides, and high-density lipoprotein (HDL) cholesterol were measured by available enzymatic methods. Low-density lipoprotein (LDL) cholesterol concentrations were estimated using the Friedewald formula. All subjects underwent a 75-g oral glucose tolerance test (OGTT) with sampling for glucose insulin and glucagon, as previously described (31). Plasma glucose was measured with the glucose oxidase method. HbA_1c_ was measured with high-performance liquid chromatography using a National Glycohemoglobin Standardization Program and standardized to the Diabetes Control and Complications Trial reference assay by a certified automated analyzer (HPLC; HLC-723G7 hemoglobin HPLC analyzer Tosoh Corp.) (normal range 4.25–5.9 % (23–41 mmol/mol)) (32).

Plasma glucagon was measured using a RIA kit (Millipore Corporation) according to the manufacturer’s instructions. For the analysis of glucagon, blood was withdrawn into chilled tubes containing heparin plus aprotinin (500 kIU/ml blood; Trasylol; Bayer, Leverkusen, Germany) and stored at −80°C until analysis.

### Calculations

Insulin resistance was estimated using HOMA-IR calculated as previously described (33). The relative glucagon suppression during the first 30 min (early) after oral glucose administration was calculated as follows (1 − (glucacon_30 min_/glucagon_0 min_)) × 100%. Similarly, the suppression of glucagon from 30 to 120 (late) was calculated as (1 – (glucagon_120 min _/ glucagon_30 min_)) × 100%, and glucagon suppression during the entire OGTT (overall) was calculated as (1 – (glucagon_120 min _/ glucagon_0 min_)) × 100%. Accordingly, this resulted in positive values for subjects who did suppress glucagon and negative values for those who did not suppress glucagon during the OGTT. We calculated the insulin sensitivity index (ISI_0-120_), reflecting whole-body insulin sensitivity, as follows: (75.000 mg + (glucose_0 min_ – glucose_120 min_) × 0.19 × weight) / (glucose_0 min _+ glucose_120 min _/ 2 × log (insulin_0 min _+ insulin_120 min _/ 2)) (34).

Early-phase insulin secretion was assessed by the insulinogenic index (IG30), calculated as the change of insulin divided by the change of glucose during the first 30 min of the OGTT. To evaluate the relationship between β-cell function and insulin resistance, disposition index was calculated as IG30/HOMA-IR (35).

### α-TC1 cell line and culture conditions

Mouse glucagonoma α-TC1 (clone 6) cell line was purchased from the American Type Culture Collection (ATCC, through LGC Standards S.r.l., Milan, Italy). This line was derived from an adenoma created in transgenic mice expressing the SV40 large T antigen oncogene under the control of the rat pre-proglucagon promoter. Cells were grown in DMEM (Dulbecco’s Modified Eagle Medium) with 4 mmol/l-glutamine (Sigma-Aldrich), modified to contain 16.7 mmol/L glucose, supplemented with 10% heat-inactivated dialyzed fetal bovine serum (Gibco, Thermo Fisher Scientific), 15 mmol/L HEPES, 0.1 mmol/L nonessential amino acids and 0.02% BSA under an atmosphere of 95% humidified air, 5% CO_2_ at 37°C. The medium was changed twice a week and cells were trypsinized and reseeded when 70% confluence was reached (approximately once a week).

### α-TC1-6 treatment with atorvastatin

Twenty-four hours after planting, the α-TC1-6 cells were cultured for 24 or 48 h at 37°C in complete DMEM medium in the presence or absence of 100 ng/mL atorvastatin (Sigma-Aldrich). To assess the effect of insulin, the cells were serum-starved for 24 h in medium with BSA 0.1% instead of FBS before stimulation with insulin. Stimulation with 10^−9^ M insulin (Sigma-Aldrich) was performed for 5 min or 2 h for the studies on insulin signaling and glucagon secretion respectively and for 24 h for the studies on glucagon gene expression as previously described (28, 36).

### Glucagon secretion from α-TC1-6

α-TC1-6 cells were plated into 6-well plates (30 × 10^4^ cells/well) and cultured as described above. At the end of the culture period, cells were washed and incubated for 2 h in Krebs-Ringer buffer (KRB) containing 16.7 mmol/L glucose and 0.5% BSA (pH 7.4) in the presence or absence of insulin 10^−9^ M. The samples were collected in vials containing aprotinin (0.1 mg/mL) and kept frozen at −20°C until the subsequent analysis. The cells were lysed in Radio-Immunoprecipitation Assay (RIPA) buffer and the lysates were analyzed for total protein content to check the number of cells. Glucagon analysis was carried out using a glucagon ELISA kit (Mercodia Glucagon; Uppsala, Sweden) according to the manufacturer’s instructions.

### Cell lysis, immunoprecipitation and Western blot analysis in α-TC1-6 cells

At the end of the culture period, proteins were extracted from α-TC1-6 cells with ice-cold RIPA lysis buffer. For insulin receptor (IR) phosphorylation analysis, immunoprecipitation was performed as previously described (37). Protein A-Sepharose (GE Healthcare Life Sciences, Uppsala, Sweden) was incubated with 2–4 μg of the IR β-subunit (IR-β) antibody (Santa Cruz Biotechnology), at 4°C under constant rotation for 2 h and then overnight with cell lysates. Immunoprecipitates were subjected to SDS-PAGE and then analyzed by immunoblotting with an anti-phospho-IR β (Tyr1150/1151) (Cell Signaling Technology).

For Western blot analysis, cell lysates were analyzed as previously described (38). Proteins of interest were detected with the following specific antibodies: anti-phospho-IRS-1 (Tyr612) and anti-total IRS-1 (Santa Cruz Biotechnology); anti-phospho-AKT (Ser473) and anti-total AKT (Cell Signaling Technology); anti β-Actin (Sigma-Aldrich).

All of the immunoblot signals were visualized using the Odyssey Fc System infra-red scanner (LI-COR Biosciences, Lincoln, NE, USA) and were subjected to densitometric analyses using Odyssey software Image studio Lite, version 5.2 as previously reported (39).

### mRNA isolation and real-time PCR analysis

Total RNA was extracted with TRIzol reagent (Thermo Fisher Scientific) according to the manufacturer’s instructions and quantified by spectrophotometry.

Quantitative Real-time PCR was performed using Power SYBR^®^ Green RNA-to-CT™ 1-Step Kit (Thermo Fisher Scientific); briefly, 50 ng of total RNA was retro-transcribed and amplified in a single step according to the manufacturer’s protocol. Proglucagon (*Gcg*, NM_008100*)* gene expression was evaluated and glyceraldehyde-3-phosphate dehydrogenase *(Gapdh*, NM_001289726*)* was used as reference gene. Each experiment was performed in biological triplicate and gene expression changes were analyzed with the 2^−ΔΔ^*^C^* ^t^ method.

### Statistical analysis

We based the power calculation on previous studies examining overall glucagon suppression among patients with altered glycemic homeostasis and control subjects; the level of significance (α) was set to 5% and power (1-β) to 80% (34). The estimated sample size was 21 subjects for each group. Statistical analyses included the unpaired *t* test and ANOVA followed by *post hoc* analysis of significance (Bonferroni test) for continuous variables and χ^2^ test for non-continuous variables. A *P* value less than 0.05 was considered statistically significant. Partial eta-squared was calculated to determine the magnitude of change, with an effect size (ES) of 0.01–0.05 considered small, 0.06–0.13 medium and ≥0.14 large.

The data are presented as the means ± s.d.s for normally distributed variables or as medians with corresponding 95% CI. The distributional characteristics of each variable were assessed by the Kolmogorov–Smirnov test. When necessary, numerical variables were logarithmically transformed to reduce skewedness. Pearson’s correlation was used to correlate ISI_0-120_ with early and overall glucagon suppression.

To identify variables independently associated with early and overall glucagon suppression, we used two multivariate regression models. The first model included age, sex, basal glucagon levels and statin therapy. The second model included variables reaching statistical significance in the first model and fasting glucose, 30-min glucose, fasting insulin, 30-min insulin, DI and ISI_0-120_ for early suppression; fasting glucose, 120-min glucose, fasting insulin, 120-min insulin, DI and ISI_0-120_ for overall suppression. The variance inflation factor was used to check for the problem of multicollinearity among the predictor variables in multiple regression analysis.

The study was approved by ‘Catania 2’ Ethical Committee and informed consent was obtained from each participant.

## Results

The study population (50 subjects) was divided into two groups based on atorvastatin therapy: 26 patients with prediabetes and statin therapy (statin therapy group) and 24 patients with prediabetes without statin therapy (controls). Clinical characteristics of the study subjects are shown in Table 1. There were no differences between patients with statin therapy compared with controls with respect to anthropometric and metabolic characteristics except for lower total and LDL cholesterol levels, as expected (168.7 ±522.4 vs 199.8 ± 32.5 (*P *< 0.001, ES = 0.16) and 99.9 ± 23.2 vs 131.6 ± 30.5 (*P *< 0.001, ES = 0.25), respectively). Subjects on statin therapy exhibited lower ISI_0-120_ compared to controls (34.9 (31.6; 46.1) vs 44.5 (43.1; 57) (*P *= 0.04, ES = 0.14)).

**Table 1:** Clinical characteristics of the study population on statin therapy. Data are presented as mean ± s.d. or median (95% CI).

	Controls (*n = *24)	Patients with statin therapy (*n *= 26)	*P*	Effect size
Age (years)	53.7 ± 6.6	54.3 ± 5.5	0.69	0.03
BMI (kg/m^2^)	30 ± 4.2	30 ± 3.9	0.9	0.01
Waist circumference (cm)	101.8 ± 11	101.8 ± 9.5	0.86	0.01
Systolic BP (mmHg)	124.7 ± 15.2	126 ± 10.9	0.7	0.00
Diastolic BP (mmHg)	79.7 ± 10.1	79.2 ± 6.1	0.91	0.00
HbA_1c_ (%)	6 ± 0.7	6 ± 0.5	0.78	0.05
Fasting glucose mg/dL	99.1 ± 10.8	102.1 ± 12.8	0.42	0.00
30-min glucose mg/dL	143.3 ± 31.1	153 ± 41.8	0.52	0.01
120-min glucose mg/dL	155.4 ± 34.1	159.1 ± 31.5	0.65	0.71
Fasting insulin microu/mL	6.5 (6.85; 12.3)	7.3 (6.22; 10.1)	0.56	0.02
30-min insulin microu/mL	33.7 (30.5; 48.2)	30.2 (27.3; 43.8)	0.48	0.01
120-min insulin microu/mL	43.4 (43.4; 86.5)	62.5 (37.1; 80)	0.18	0.04
Total cholesterol (mg/dL)	199.8 ± 32.5	168.5 ± 22.4	0.001	0.16
HDL cholesterol (mg/dL)	44.1 ± 13.4	41.5 ± 10.5	0.64	0.03
Triglycerides (mg/dL)	78.7 (78.7; 122.2)	143 (117; 168.8)	0.15	0.19
LDL cholesterol (mg/dL)	131 ± 30.5	99.9 ± 23.2	0.001	0.25
Sex (M/F)	12/9	12/10	0.9	0.00
HOMA-IR	1.4 (0.77; 2.03)	1.6 (0.79; 2.41)	0.34	0.05
ISI_0-120_	44.5 (43.1; 57)	34.9 (31.6; 46.1)	0.04	0.14
IG30	0.78 (0.54; 1.26)	0.59 (0.34; 0.52)	0.19	0.11
DI	0.36 (0.27; 0.99)	0.26 (0.17; 0.27)	0.21	0.15

BMI, body mass index; BP, blood pressure; DI, disposition index; HbA_1c_, glycated hemoglobin; HDL, high-density lipoprotein; HOMA-IR, homeostasis model assessment-insulin resistance; ISI_0-120_, insulin sensitivity index 0-120; IG30, insulinogenic index; LDL, low-density lipoprotein.

**Figure 1:**
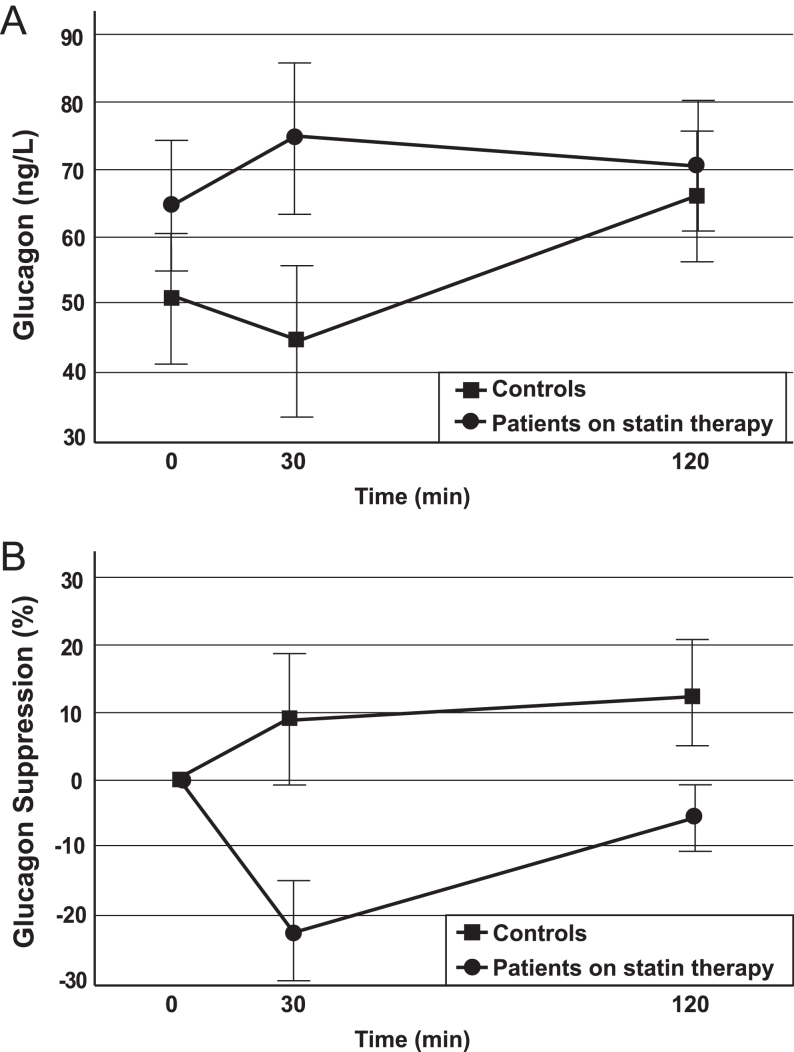
Plasma glucagon and glucagon suppression during the OGTT. Plasma glucagon levels (A) and relative glucagon suppression (B) in controls (filled squares) and patients on statin therapy (filled circles), during OGTT. Data are reported as means ± s.d. Glucagon suppression 0–30' vs controls; *P *< 0.05 Glucagon suppression 0–120' vs controls; *P *< 0.05.

Absolute glucagon levels and the percentage of glucagon suppression during the OGTT in these groups are shown in Table 2 and Fig. 1. Although without statistical significance, basal and 30- min plasma glucagon levels were slightly higher in the statin therapy group compared with controls (67 (53.7; 79.2) vs 48 (29.3; 62.2), (*P *= 0.21, ES = 0.14) and 77 (55.2; 90.1) vs 41.1 (33.3; 64.6), (*P *= 0.1, ES = 0.1) respectively); no differences were found in 120-min glucagon levels between the two groups.

**Table 2:** Plasma glucagon characteristics of the study population on statin therapy. Data are presented median (95% CI).

	Controls (*n = *24)	Patients on statin therapy (*n *= 26)	*P*	Effect size
Fasting glucagon (ng/L)	48 (29.3; 62.2)	67 (53.7; 79.2)	0.21	0.05
30-min glucagon (ng/L)	41.1 (33.3; 64.6)	77.3 (55.2; 90.1)	0.1	0.1
120-min glucagon (ng/L)	66.6 (46.7; 78)	70.4 (59.7; 85.8)	0.77	0.00
Glucagon suppression (%)				
0–30 min	8.8 (−6.7; 16.9)	−25 (−27.7; 3.7)	0.003	0.15
30–120 min	13.5 (−6.1; 17.5)	−8.3 (−20.7; 12.7)	0.1	0.11
0–120 min	20.5 (2.9; 29)	2 (−20; 10.3)	0.01	0.18

Furthermore, subjects on statin therapy showed a reduced early (0–30 min) and overall (0–120 min) glucagon suppression during the OGTT (−25 (−27.7; 3.7) vs 8.8 (−6.7; 16.9), (*P *= 0.003, ES 0.15) and 2 (−20; 10.3) vs 20.5 (2.9; 29), (*P *= 0.01, ES = 0.18)). Although without statistical significance, late glucagon suppression (30–120 min) was slightly lower in the statin therapy group compared with controls (8.3 (−20.7; 12.7) vs 13.5 (−6.1; 17.5), (*P *= 0.1, ES 0.11)) (Table 2 and Fig. 1).

In multivariate regression analysis (Table 3), we investigated the association of early and overall glucagon suppression with statin therapy using separate models.

**Table 3:** Multiple regression analysis evaluating early (0–30) and overall (0–120) glucagon suppression as dependent variables.

	Mean difference	95% CI	*P* value
Early glucagon suppression_0-30_			
Model 1*			
Statin therapy	−16.7	−46.5; −2.9	0.01
Model 2**			
Statin therapy	−16.7	−52.5; −2.9	0.03
Fasting insulin	−5.3	−8.2; −2.3	0.007
Overall glucagon suppression_0-120_			
Model 1*			
Sex	26.4	2.8; 49.9	0.02
Statin therapy	−27.7	−52.5; −2.9	0.04
Model 2**			
Fasting insulin	−3.5	−5.7; −1.3	0.03
ISI_0-120_	0.47	0.08; 0.596	0.01

*Model 1 was adjusted for age, sex, basal glucagon and statin therapy

**Model 2 was adjusted for variables reaching statistical significance in the first model and fasting glucose and 30-min glucose, fasting insulin, 30-min insulin, DI and ISI_0-120_ for early glucagon suppression; fasting glucose, 120-min glucose, fasting insulin, 120-min insulin, DI and ISI_0-120_ for overall glucagon suppression.

Early glucagon suppression (0–30 min) exhibited a significant correlation with statin therapy in the first model (Table 3). In the second model the variables that remained significantly associated with early glucagon suppression were statin therapy and fasting insulin. Overall glucagon suppression (0–120) was associated with sex, ISI_ 0-120_ and statin therapy in the first model, and with fasting insulin and ISI _0-120_ in the second model (Table 3). According to our hypothesis, regression analysis showed a significant association between ISI _0-120_, early and overall glucagon suppression (*r* = 0.33, *P *= 0.001 and *r* = 0.38, *P *= 0.001, respectively).

### Atorvastatin treatment affected insulin suppression of glucagon secretion in α-TC1 cells

To investigate the molecular mechanisms involved in the alterations induced by statin therapy, we studied the direct effect of atorvastatin on glucagon secretion, IR function and insulin signaling pathway, using the clonal α-TC1-6 cell line, a well-validated *in vitro* model of pancreatic glucagon-secreting α-cells (40).

We first investigated acute insulin inhibition of glucagon secretion in control cells and in cells that had been chronically pre-exposed to atorvastatin (100 ng/mL for 24 or 48 h).

**Figure 2:**
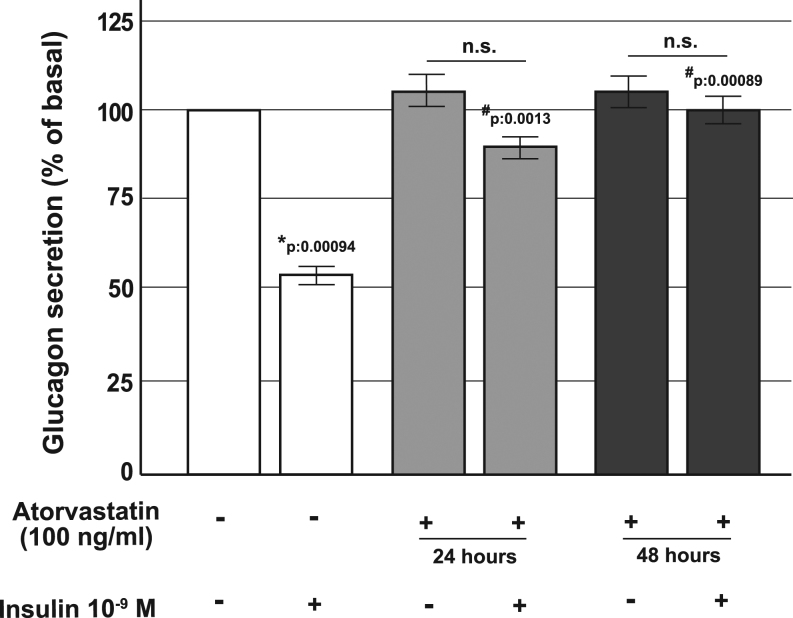
Effect of atorvastatin on glucagon secretion in α-TC1 cells. Acute glucagon secretion in control cells and in cells pre-exposed to 100 ng/mL of atorvastatin for 24 or 48 h and/or insulin 10−9 M (baseline secretory rate at 16.7 mM glucose in absence of insulin: 560 fmol/µg proteins/h ± 15 in 1 h); *P < 0.05, vs control in absence of insulin stimulation; ^#^*P* < 0.05 vs insulin stimulated control; n.s. not significant (one-way ANOVA followed by Bonferroni test, *n* = 4).

After the 24 h of serum starvation, cells were washed and cultured for 2 h in KRB in the presence or absence of atorvastatin (100 ng/mL) and/or insulin 10^−9^ M; at the end of the incubation, glucagon levels were measured. As shown in Fig. 2, under control conditions, glucagon secretion was significantly inhibited by insulin and the glucagon concentration in the medium was significantly reduced (560 fmol/µg of proteins/h ± 15 vs 287 ± 8.7 fmol/µg of proteins/h, *P* = 0.00094). In contrast, the inhibitory effect of insulin was markedly reduced in cells pre-exposed to atorvastatin.

### Atorvastatin treatment impaired insulin signaling in α-TC1 cells

The binding of insulin to its tyrosine kinase receptor (IR) engages a cascade of intracellular phosphorylation events, including tyrosine phosphorylation of the insulin receptor substrate-1 (IRS-1) protein and activation of the serine-threonine protein kinase (AKT). To further investigate the molecular mechanisms by which atorvastatin altered the inhibitory action of insulin on glucagon secretion, we analyzed the intracellular insulin pathway in α-TC1-6 cells chronically (24 or 48 h) treated with atorvastatin 100 ng/mL.

**Figure 3:**
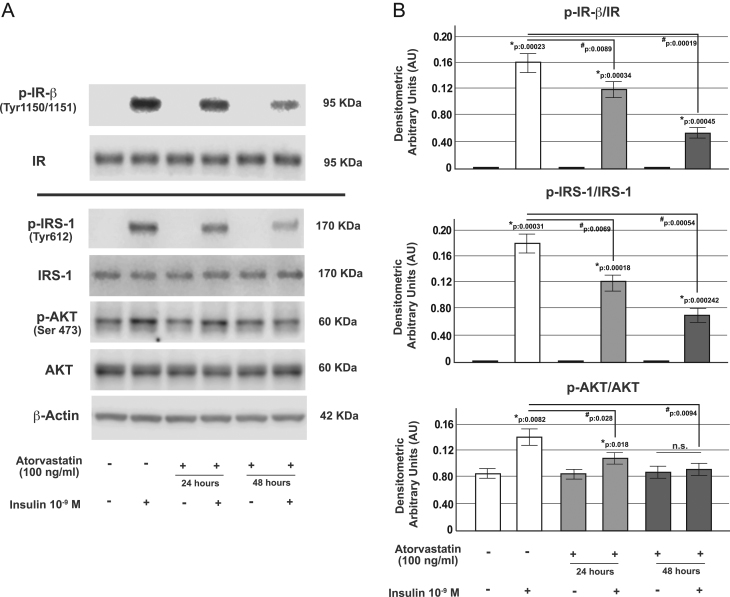
Effect of atorvastatin on IR phosphorylation and IRS-1/AKT pathway in α-TC1-6 cells. (A) Representative immunoblot from control cells and cells pre-exposed to atorvastatin (100 ng/mL for 24 or 48 h) and acutely stimulated with insulin (10^−9^ M for 5 min) for (from the top to bottom): immunoprecipitation of the total IR (Tyr1150/1151-β subunit) (p-IR-β) and total IR; p-IRS-1 (Tyr612) and total IRS-1 (IRS-1); p-AKT (Ser 473) and total AKT (AKT); β-Actin; Panel B: corresponding densitometric analysis. * *P* < 0.05 vs controls in the absence of insulin stimulation; # *P* < 0.05 vs insulin-stimulated control group; n.s. not significant (one-way ANOVA followed by Bonferroni test, *n* = 3).

As shown in Fig. 3, under control conditions, insulin stimulation (10^−9^ M for 5 min) caused a significant increase of IR-β, IRS-1 (Tyr612) and AKT (Ser473) phosphorylation; conversely, in atorvastatin pre-exposed cells, the insulin activation of these proteins was significantly reduced, as evidenced by Western blot analysis.

### Effects of atorvastatin on proglucagon gene expression

Through the activation of AKT, insulin negatively regulates proglucagon gene (*Gcg*) expression (36) in pancreatic α-cells.

**Figure 4:**
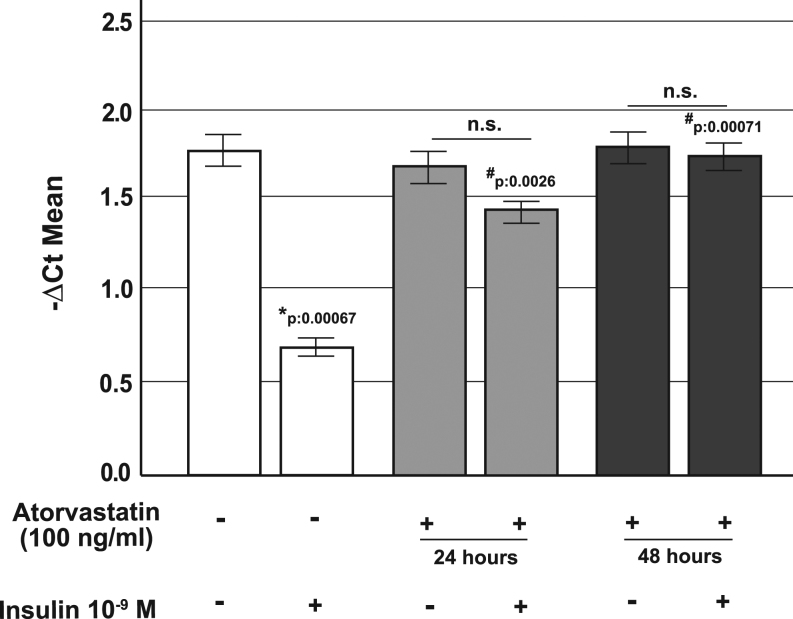
Effect of atorvastatin on proglucagon gene expression in α-TC1 cells. Real-time analysis for proglucagon gene expression in control cells and in cells pre-exposed to atorvastatin (100 ng/mL for 24 or 48 h) and/or insulin (10^−9^ M for 24 h). *P < 0.05, vs control in absence of insulin stimulation; #*P* < 0.05 vs insulin stimulated control; n.s. not significant (1-way ANOVA followed by Bonferroni test, *n* = 3). Data are expressed as ∆Ct multiplied by −1.

To further investigate the mechanisms by which atorvastatin affects glucagon secretion we examined the insulin action on *Gcg* gene expression. As shown in Fig. 4, while under control conditions proglucagon gene expression was significantly (*P* = 0.00067) inhibited by insulin treatment (10^−9^ M for 24 h), in cells pre-exposed to atorvastatin (100 ng/mL for 24 or 48 h), the insulin-induced inhibition of *Gcg* gene transcription was almost abolished.

## Discussion

Recent clinical data have shown that chronic statin therapy is associated with an increased risk of type 2 diabetes mellitus (3, 4, 5, 13); however, the physiopathologic mechanisms behind this association are not fully understood. In this study, we explored glucagon suppression and its relationship with insulin resistance in subjects on atorvastatin therapy at high risk for type 2 diabetes.

We then studied the direct action of atorvastatin on pancreatic α-cells *in vitro*; we investigated insulin-mediated glucagon suppression, insulin signaling pathway and, finally, proglucagon gene expression in glucagon-producing αTC1-6 cells chronically exposed (24 or 48 h) to atorvastatin (100 ng/mL). Atorvastatin was chosen because, together with simvastatin, it is the most commonly used statin in clinical practice (41).

The main finding of this study was that prediabetic subjects on atorvastatin therapy exhibited reduced early and overall glucagon suppression compared with controls. Moreover, we found similar late glucagon suppression (from 30 to 120 min) between the two groups during OGTT. Impaired glucagon suppression in response to glucose or meal challenges was described in previous studies in subjects with prediabetes and type 2 diabetes (42) and was reported as a key pathophysiologic feature of type 2 diabetes and altered glucose tolerance (43). However, to the best of our knowledge, the association of statin therapy with glucagon suppression in prediabetic subjects has not been evaluated so far.

Furthermore, we found that overall glucagon suppression was associated with lower insulin sensitivity; taken together, these results support our hypothesis that increased circulating levels of glucagon, together with increased insulin levels, are tightly coupled to a reduction of insulin sensitivity in the early stages of glucose dysregulation (34). According to previous data, the body promotes physiological adaptations to insulin resistance by also increasing the suppression of glucagon levels in response to glucose, underlying the role of alpha cells during the development of insulin resistance (44). The changes in glucagon secretion should constitute alpha cell adaptations in line with the reduction of insulin sensitivity. In statin-treated patients this mechanism may be, at least partially, impaired determining a reduced glucagon suppression in response to glucose load.

Recent literature has remarked the importance of the equilibrium based on the α- to β-cell signaling. Capozzi and Svendsen *et al*. recently investigated the intra-islet paracrine relationship and demonstrated an essential role for alpha cell in the regulation of beta cell function in different models, raising the possibility that abnormal paracrine signaling contributes to alteration in glucose homeostasis during diabetes and emphasizing a role for paracrine intra-islet α-cell actions to maintain appropriate β-cell function (45, 46). Although there is still a lack of a clear molecular explanation for the adverse effects of statin therapy on insulin sensitivity, this association has been reported in other studies: Duvniak *et al* showed that statin therapy introduction was associated with 36% increased risk of reduced insulin sensitivity after adjustment for confounding risk factors in subjects with type 1 diabetes; these findings were observed after 56 months of statin exposure (47). Furthermore, Cederberg *et al.* reported a reduction in insulin sensitivity and secretion in non-diabetic Finnish men on statin therapy; decreases in insulin sensitivity and insulin secretion were dose dependent for simvastatin and atorvastatin (7). Accordingly, our group showed that atorvastatin affected insulin release and mitochondrial metabolism due to the enhancement of oxidative stress, in a previous study performed on human pancreatic islets (21).

In the current work, we provide evidence that in α-TC1 pancreatic cells chronically (24 or 48 h) exposed to atorvastatin (100 ng/mL), the inhibitory effect of insulin on glucagon secretion was blunted and that phosphorylated forms of IR-β, IRS-1 and AKT were all reduced. In addition, we observed that in atorvastatin-treated α-cells, the insulin-induced inhibition of proglucagon gene transcription was significantly impaired. Insulin resistance induced from statin exposure has already been reported in different *in vitro* models and explained by other molecular mechanisms. In 3T3‑L1 cells and isolated rat adipocytes, statin treatment has been shown to be associated with a decreased expression of GLUT4 associated with a marked reduction in tyrosine-specific phosphorylation of IRS-1 in response to insulin (22, 48, 49). Moreover, it has been observed that inhibition of HMG-CoA reductase affected membrane concentrations of small GTP‑binding proteins such as Rab4 and RhoA whose action is essential for insulin signal transduction (50). Kain *et al*. (51) had shown that statin treatment blocked insulin-mediated glucose uptake by a free fatty acid accumulation-mediated pathway in L6 myotubes; similar results have been found in primary cultured rat cardiomyocytes (52). Our work focused on the role of glucagon secretion and pancreatic α-cell function during insulin resistance states specifically induced by statin exposure. Accordingly, we found that chronic treatment with atorvastatin induced insulin resistance of the IR/IRS-1/AKT pathway and through this mechanism increased proglucagon gene expression and glucagon secretion. Expression of the IR has been described in pancreatic α-cells (53), and its expression is similar to other major insulin target tissues, such as the liver or skeletal muscle (54). Based on our results pancreatic α-cells could be reasonably added to the list of insulin-sensitive tissues that can become irresponsive and resistant during statin exposure.

It should be noted that also the contribution of glucagon on insulin sensitivity is relevant in several tissues; indeed, there is evidence that the liver may be hypersensitive to the stimulatory effect of glucagon in hepatic gluconeogenesis (55) and that glucagon induces postprandial peripheral insulin resistance through reducing hepatic glutathione levels in an animal models (56).

There were several limitations of this study. First, this was a cross-sectional study and a causal relationship between changes in glucagon suppression and statin therapy cannot be established; the physiology of glucagon is complex and involves an intricate series of mechanisms and oral glucose can stimulate gastrointestinal hormone secretion, which may also have an impact on glucagon secretion. Second, although we were able to show a significant association between variables, the sample size was relatively small; it may be possible to evidence other differences between the two groups in a larger population. Finally, we took into consideration only subjects with prediabetes in therapy with atorvastatin; thus, our results leave the question of whether the findings can be applied to those patients in therapy with other statins open.

Our molecular data were obtained using an *in vitro* model. Like all studies performed on cell lines, αTC1-6 cells cannot fully mimic α-cells *in vivo*; however, αTC1-6 cells possess an advantage over primary islets as they represent a homogeneous cellular population and for this reason have been extensively used to study glucagon secretion and proglucagon gene expression (40, 57).

In conclusion, our data demonstrated that prediabetic patients undergoing statin therapy exhibit low glucagon suppression after glucose load; furthermore, in these subjects impaired glucagon suppression was associated with lower insulin sensitivity.

Our data strongly suggest that statins exert their detrimental effects on insulin sensitivity and glucagon suppression by directly affecting IR activation and the related intracellular signaling cascade at the level of the pancreatic α-cell. As a result, pancreatic α-cell become unable to adequately respond to the action of insulin and suppress glucagon release.

Our results may have important clinical implications because they reveal a new molecular aspect behind the deregulation of insulin sensitivity secondary to statin exposure and provide a link between statin treatment, impaired insulin sensitivity and glucagon hypersecretion.

## Declaration of interest

The authors declare that there is no conflict of interest that could be perceived as prejudicing the impartiality of the research reported.

## Funding

This work was supported by 2016/2018 Department Research Plan of University of Catania, Italy, Department of Clinical and Experimental Medicine (project #A).
